# Supplementary material for: Information dynamics and the emergence of high-order individuality in ecosystems
Source: Commun Biol. 2025 Aug 15;8:1231. doi: 10.1038/s42003-025-08619-2 (PMC12356948; doi:10.1038/s42003-025-08619-2)
Supplement: Supplementary file 2 — Supplementary Information [file 42003_2025_8619_MOESM2_ESM.pdf]

# Information dynamics and the emergence of high-order individuality in ecosystems

## Supplementary Information

## A Species fitness and inter-species distance near error-threshold

To better understand the effect of the mutation rate, we explored its effect on the reproduction rate and distance between extant species in the genome space. Note that the reproduction rate is quantified using the $p_{off}$ of each species, based on the inter-species interactions. Results show that low mutation rates support the existence of a handful of very fit species with a cloud of mutants around them (see Figure A.1). In contrast, higher mutation rates allow more non-trivial combinations of species existing near the error-threshold, which is confirmed by the increasing Hamming distance among the species and the decreasing gap of fitness between the top few species and the others.

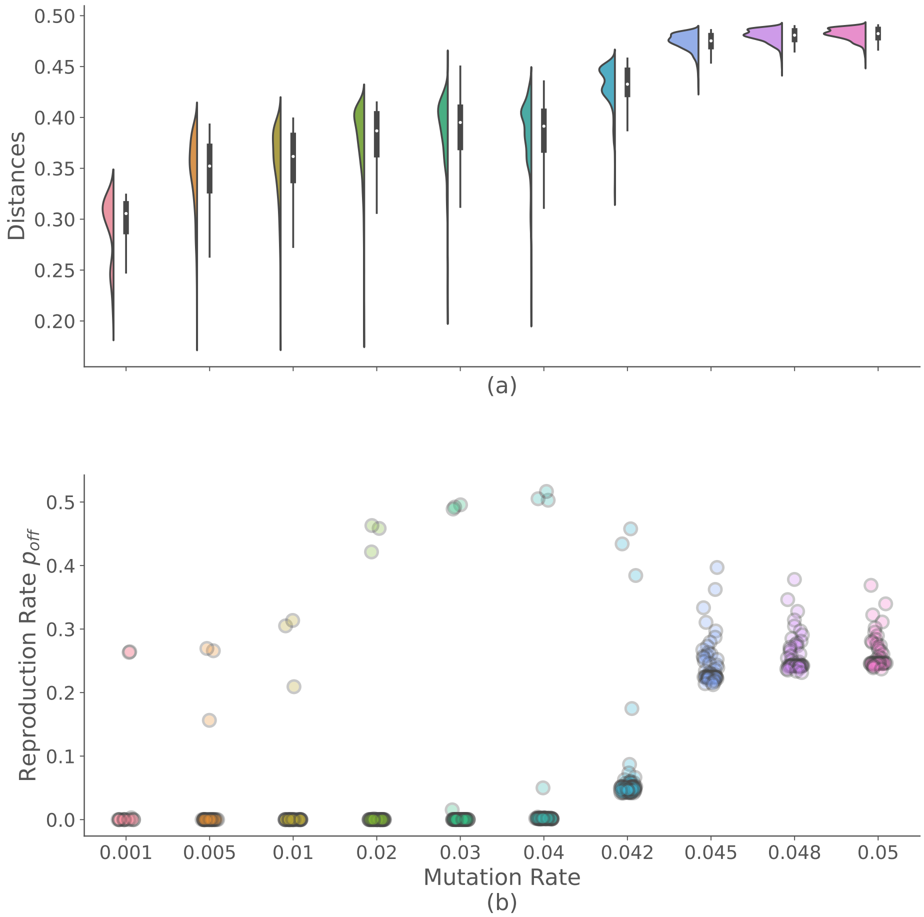


**Figure A.1.** As mutation rate increases, the existing species of the system become more genetically distant from each other (a), as shown by the distribution of species in the genome space. Figure (b) shows the reproduction probability ($p_{off}$) of each existing species as a scatter plot. It can be seen that for low mutation rates, a few (highly populated) existing species reproduce at a significantly higher rate than the other existing species.

## B Alternative individuality scores

As mentioned in the methods section, we focus on the organismal individuality as defined in[1]. However, we replicated the analysis for the colonial individuality scores and found a very similar hierarchical organization as reported in the results above.

As seen in figure B.1 and B.2, both colonial and environment-determined individuality scores exhibit higher individuality scores for scales 3-9 and intermediate mutation rates. This aligns with the results presented for the organismal individuality in the main results (see Figure 4). However, this peak flattens as we approach error-transition. Near this threshold, individual species have the highest colonial and environment-dependent individuality scores.

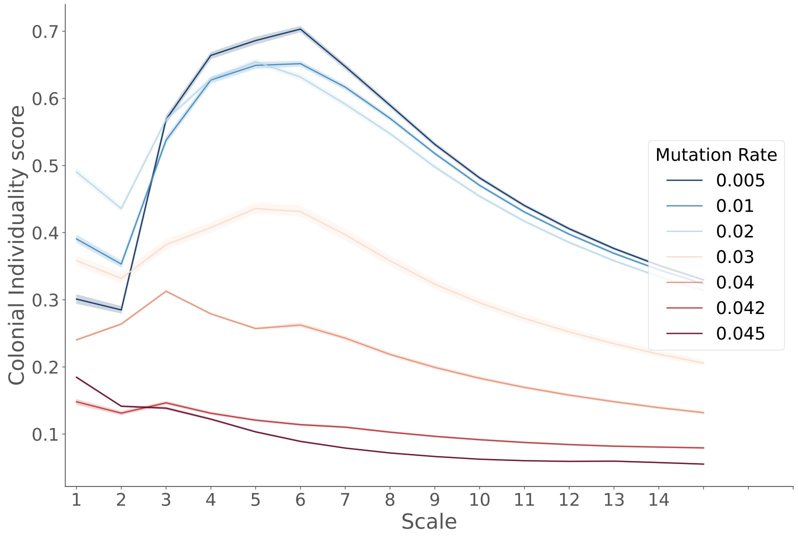


**Figure B.1.** Colonial individuality scores for different scales of organization and mutation rates.

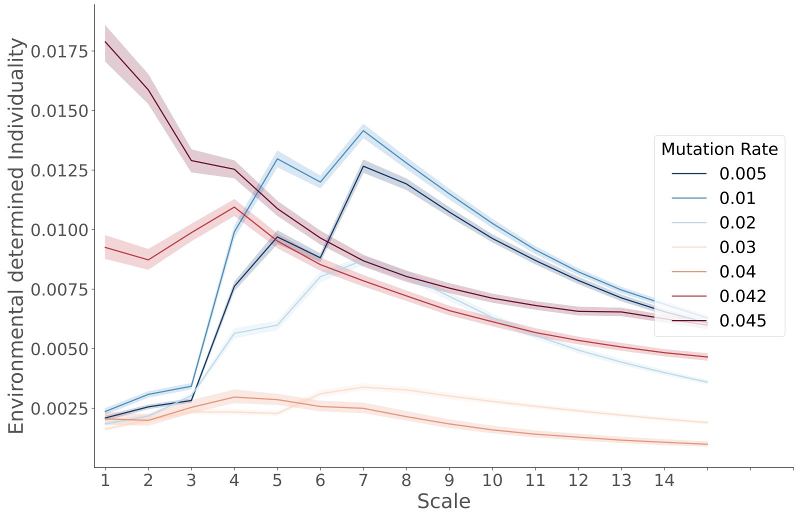


**Figure B.2.** Environment-dependent individuality scores for different scales of organization and mutation rates.

## C Non-Stationary dynamics

The Tangled Nature model exhibits time-dependent non-stationary dynamics, such that the inter-transition intervals decay slowly over time. As discussed before, the system evolves into more stable configurations over time, so the number of mass extinction events goes down in time. The population and diversity also vary logarithmically in generational time. Therefore, the probability distribution of any given species or the total population changes with time. Therefore, we estimate probability distributions for each timestep across an ensemble of simulations to estimate information-theoretic measures. This method enables time-resolved estimation of mutual information by estimating probability distributions at each timestep across the ensemble[2]. For the current analysis, we use over 10,000 simulations for every mutation rate to estimate information-theoretic measures. This procedure is regularly applied in neuroscience studies where brain activity varies in response to stimulus[3]. Deploying the ensemble method also allows us to observe the evolution of information measures in time. To study long-term behaviour, we sub-sampled the data and calculated the measures for every 1000th and 1001st generation. This enables us to calculate information measures like Transfer entropy and individuality scores with time delay 1 for every 1000th generation C.1. Such an ensemble of time series enables us to estimate information measures for non-stationary time series. The method is briefly described in the figure below C.2. For more details, please refer to the papers discussing the method in detail (Gomez et al.[2] and Section on Event-Related Design on page 7 of Ince et. al.[3]).

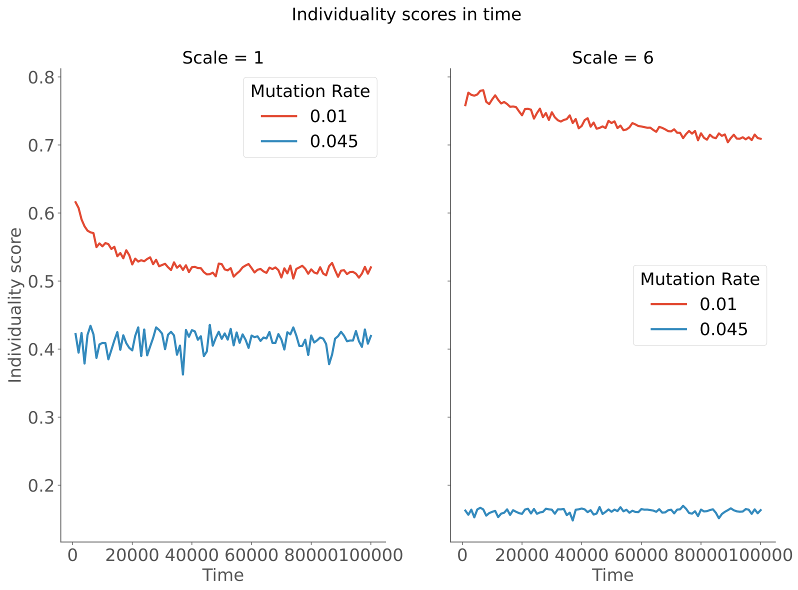


**Figure C.1.** Individuality Scores estimated at scales 1 and 6 using the Ensemble MI method at every 1000th generation for a 100,000 generation. It can be seen that the individuality scores stabilise over generations.

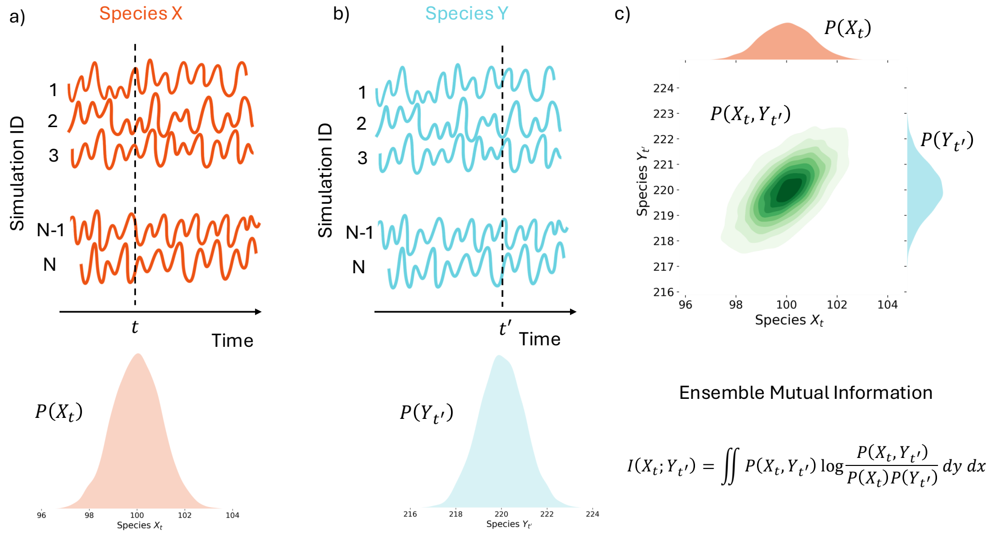


**Figure C.2.** Here we discuss the Ensemble Mutual Information approach in the context of the Tangled Nature Model. Suppose we wish to compute MI between two species (X and Y) with populations that change over time. Across the N simulations, initialised with different initial conditions, we can estimate the probability distribution of the population of (a) species X and (b) species Y at any given timestep in $t$ and $t'$ respectively, across the different values of populations across the ensemble. (c) Similarly, the joint distribution of populations of X and Y can also be estimated. The estimated marginal and joint probability distributions can then be used to compute the Ensemble Mutual Information.

## D Normalization of Information measures

Since the individuality scores are estimated at different scales, the amount of total information contained in the groups of variables of different dimensions increases with system size. Therefore, the mutual information between the current and future state of groups of variables is bounded by their joint entropy. As described in the previous section, the information measures are estimated for values across the ensemble of simulations. This leads to normally distributed values of the population of

species across different simulations at each time step.

For Gaussian random variables $X$ of dimension $K$, mean $\mu$, and covariance $\Sigma$, the joint entropy can be written in closed form as,

$$H(X) = \frac{1}{2}\ln|\Sigma| + \frac{K}{2}(1+\ln 2\pi)$$

The maximum value of the entropy of a multivariate Gaussian with dimension $K$ is for the case of uncorrelated variables where $\Sigma$ is an identity matrix such that, $|\Sigma| = 1$. So the maximum entropy for a Gaussian with dimension $K$ can be written as,

$$H(X) = \frac{K}{2}(1+\ln 2\pi)$$

The above equation provides an upper bound on the MI values estimated for the individuality scores and is directly proportional to $K$. Therefore, we use the group size as the normalizer for individuality scores.

# E Interaction structure and individuality

To understand the role of interaction structure in supporting information individuality, we looked at the average interaction structure among subsets of 6 species with the top percentile of individuality scores for different mutation rates. To clarify, the complete interaction matrix (interactions between all possible species) is fixed initially. However, in a given simulation, only a subset of existing species survive, yielding an observed interaction structure, a subset of the whole interaction matrix. Here, we discuss the average interaction structure of $K = 6$ species observed over many simulations for the different mutation rates.

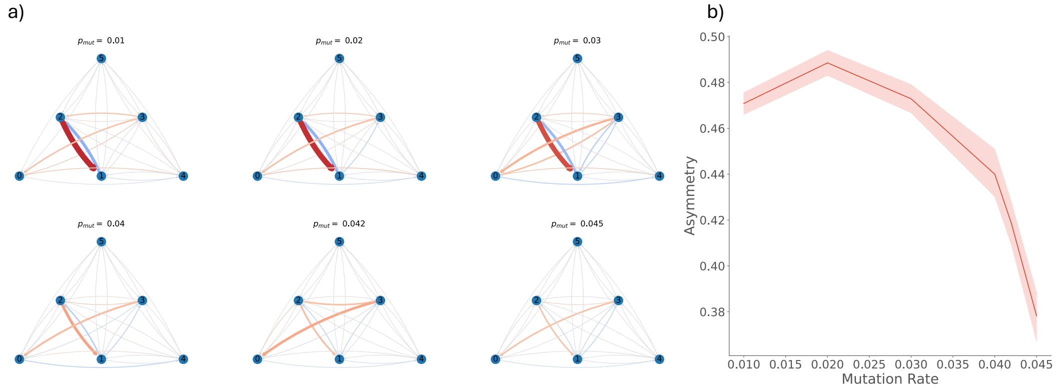


**Figure E.1.** a) Shows the average network structure of subsets of 6 species with individuality scores in the top percentile of all combinations estimated. We see a salient predator-prey interaction and high asymmetry for low mutation rates. For mutation rates in the transition region, the asymmetry and the predator-prey interaction fade away along with the higher order organization. (b) Network asymmetry is calculated using the RMS difference of the incoming and outgoing network edges.

We find that a very peculiar interaction structure exists for the mutation rates where higher-order organization is present (see Figure E.1). There is a salient predator-prey interaction, and the structure is highly asymmetric overall. However, for mutation rates in the transition region, the predator-prey interaction becomes much weaker, and the structure becomes less asymmetric. Network asymmetry is an indicator of hierarchical organization among the species.

To establish the role of interaction structure in supporting higher-order organization in the Tangled Nature model, we explored a neutral model with fixed positive-only interactions among all the species. Keeping all the other parameters of the Tangled Nature Model the same, we fix the $J$ matrix as an all-to-all interaction matrix (with the same weight of $+1$). By fixing the interaction network as a fully connected network with equal weight, we ensure that the system always ends up in a trivial interaction structure. The neutral model enables us to explore if the higher order organization still emerges if there are no balanced structures in the network to explore. For the mutation rates tested ($p_{mut} \in [0.01, 0.04]$), we found that no scales greater than 2 showed increased individuality scores as compared to scale 1 (see Figure E.2). This trend is in contrast to the higher-order organization observed in scales 3-9 in the main results (see Figure 4). These results show that a sparse interaction structure, with positive and negative feedbacks, is essential for the emergence of higher-order organization.

Another possible null model to explore the impact of interactions could be fixing all weights of the interaction matrix to $-1$. However, if an all to all-to-all interaction matrix is introduced with negative weights, it would lead to all the species in the

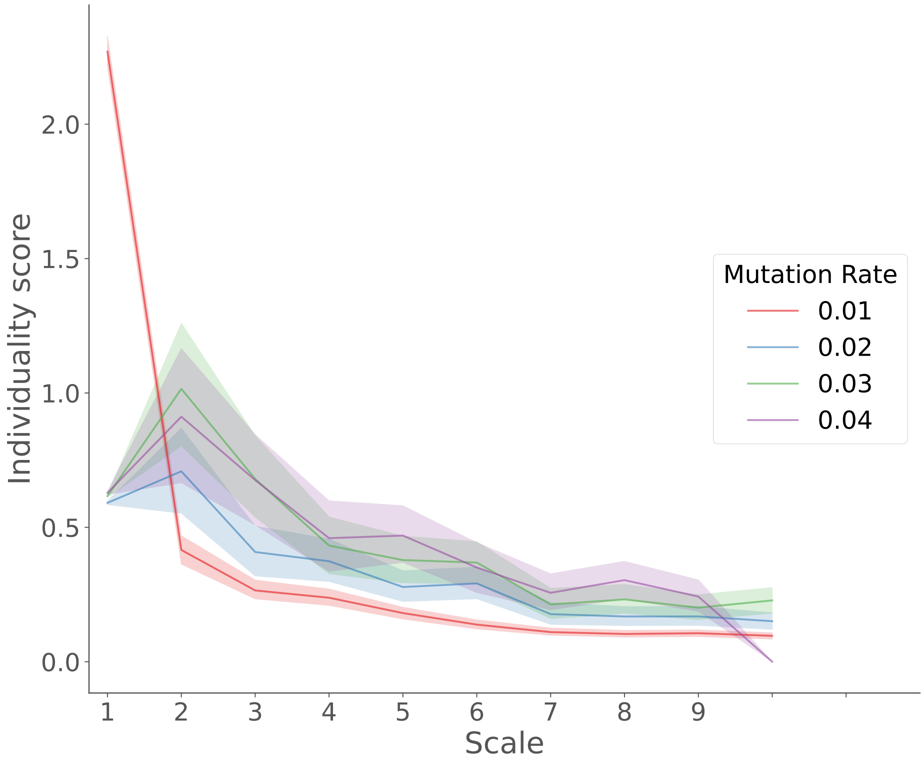


**Figure E.2.** Here we present the organismal individuality scores for the neutral model. It can be seen that as mutations increase, the scale 2 individuality is slightly higher than scale 1. However, the individuality scores decay beyond scale 2 for all mutation rates. Indicating a sparse interaction structure with positive and negative weights is necessary for higher-order organization to emerge.

ecosystem to extinction as their fitness depends on all the (negative) interactions with other species. In a sparse interaction network with negative weights, the evolution will drive the system to an uncorrelated case where none of the species interacts with the other and occupy distant spaces in the interaction network. In such an uncorrelated case, the normalized individuality score of the group of species will remain constant with increasing scale (see Appendix D).

## F Integrated information decomposition

Recently, the PID formulation for single target (see[4]) was extended to multiple targets under the Integrated Information Decomposition ($\Phi$ID)[5]. Under this extended framework, redundant, unique and synergistic information shared between multiple sources and targets can be separately defined. Thus, for a system with two parts, 16 information atoms are now defined for the respective source and target. For example, $\text{Unq}^1 \rightarrow \text{Unq}^2$ shows unique information transferred from variable 1 in the source to variable 2 in the target. Similarly, $\text{Unq}^2 \rightarrow \text{Syn}$ shows the information that was unique to variable 2 in the source and is now synergistically available in the target. Exact mathematical definitions of these atoms can be found in the $\Phi$ID paper[5].

This new framework for quantifying temporal information relationships in a system also helps in addressing some of the issues with the $\Phi$ measure of functional segregation and integration discussed in the methods section (see equation 6). Firstly, the original $\Phi$ measure provided the same numerical value for fundamentally different information processes. By decomposing $\Phi$ into information atoms, these processes can now be differentiated. This decomposition can be written as follows,

$$\begin{aligned}\Phi = {} & \text{Syn} \rightarrow \text{Syn} + \text{Syn} \rightarrow \text{Unq}^i + \text{Syn} \rightarrow \text{Red} \\ & + \text{Unq}^i \rightarrow \text{Syn} + \text{Red} \rightarrow \text{Syn} + \text{Unq}^i \rightarrow \text{Unq}^j \\ & - \text{Red} \rightarrow \text{Red}\end{aligned} \tag{1}$$

Secondly, the negative double redundancy term ($\text{Red} \rightarrow \text{Red}$) yields $\Phi$ negative for systems with a lot of redundant information flow. So by adding back this term and keeping the synergistic and transfer terms intact, a non-negative measure of information integration $\Phi_R$ can be defined[6],

$$\begin{aligned}\Phi &= \text{Synergy} + \text{Transfer} - \text{Redundancy} \\ \Phi_R &= \Phi + \text{Redundancy} \\ \Phi_R &= \Phi + \text{Red} \rightarrow \text{Red}\end{aligned} \quad (2)$$

Systems with high redundancy among the parts often show negative values for $\Phi$. This is relevant for the Tangled Nature model as evolutionary models often have a high degree of redundancy. We found this to be true in the case of the TaNa model, as seen from the figure below F.1.

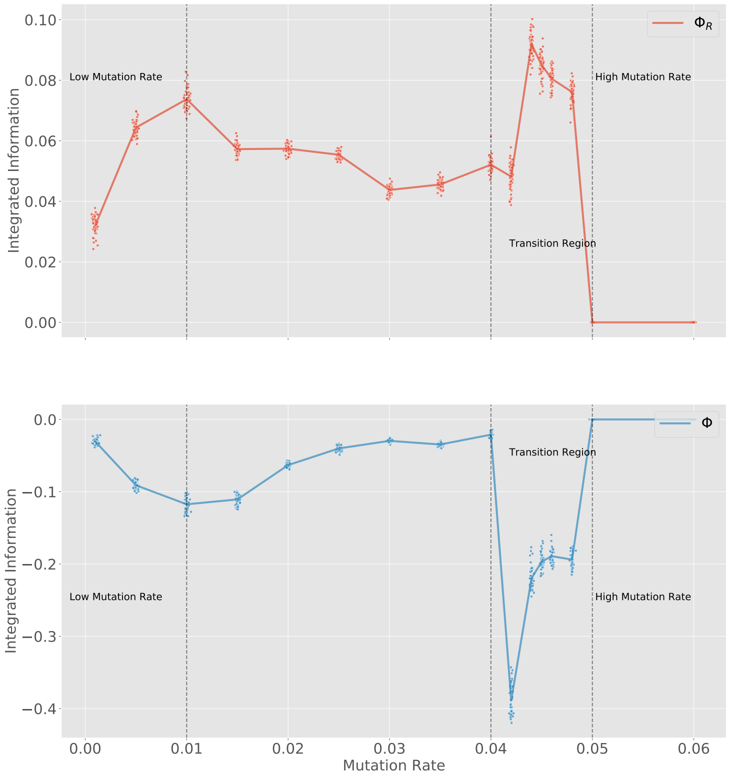


**Figure F.1.** Comparison of $\Phi_R$ and $\Phi$ measures of integrated information for varying mutation rates.

It can be seen in the figure F.1 that where a positive peak is observed for $\Phi_R$ during the error-transition region, $\Phi$ shows a negative peak. Such a negative peak would imply a lack of integration between the species and the environment. However, $\Phi_R$ confirms the contrary.

## References


1. Krakauer, D., Bertschinger, N., Olbrich, E., Flack, J. C. & Ay, N. The information theory of individuality. *Theory Biosci.* **139**, 209–223 (2020).
2. Gómez-Herrero, G. *et al.* Assessing coupling dynamics from an ensemble of time series. *Entropy* **17**, 1958–1970 (2015).
3. Ince, R. A. *et al.* A statistical framework for neuroimaging data analysis based on mutual information estimated via a gaussian copula. *Hum. brain mapping* **38**, 1541–1573 (2017).
4. Williams, P. L. & Beer, R. D. Nonnegative decomposition of multivariate information. *arXiv preprint arXiv:1004.2515* (2010).
5. Mediano, P. A., Rosas, F., Carhart-Harris, R. L., Seth, A. K. & Barrett, A. B. Beyond integrated information: A taxonomy of information dynamics phenomena. *arXiv preprint arXiv:1909.02297* (2019).
6. Luppi, A. I. *et al.* What it is like to be a bit: an integrated information decomposition account of emergent mental phenomena. *Neurosci. Conscious.* **2021**, niab027 (2021).
