# Supplementary material for: Information dynamics and the emergence of high-order individuality in ecosystems
Source: Commun Biol. 2025 Aug 15;8:1231. doi: 10.1038/s42003-025-08619-2 (PMC12356948; doi:10.1038/s42003-025-08619-2)
Supplement: Supplementary file 3 — Description of Additional Supplementary Files [file 42003_2025_8619_MOESM3_ESM.pdf]

Description of Additional Supplementary Files

File name: Supplementary Data 1

Description: Source data for generating Figures 3-7 in the main manuscript.
